# Vector Graph Assisted Pedestrian Dead Reckoning Using an Unconstrained Smartphone

**DOI:** 10.3390/s150305032

**Published:** 2015-03-02

**Authors:** Jiuchao Qian, Ling Pei, Jiabin Ma, Rendong Ying, Peilin Liu

**Affiliations:** Shanghai Key Laboratory of Navigation and Location-based Services, School of Electronic Information and Electrical Engineering, Shanghai Jiao Tong University, Shanghai 200240, China; E-Mails: andychin9@gmail.com (J.Q.); majb@live.cn (J.M.); uingrd@gmail.com (R.Y.); liupeilin@sjtu.edu.cn (P.L.)

**Keywords:** indoor localization, pedestrian dead reckoning, principal component analysis, particle filter, vector graph, smartphone sensors

## Abstract

The paper presents a hybrid indoor positioning solution based on a pedestrian dead reckoning (PDR) approach using built-in sensors on a smartphone. To address the challenges of flexible and complex contexts of carrying a phone while walking, a robust step detection algorithm based on motion-awareness has been proposed. Given the fact that step length is influenced by different motion states, an adaptive step length estimation algorithm based on motion recognition is developed. Heading estimation is carried out by an attitude acquisition algorithm, which contains a two-phase filter to mitigate the distortion of magnetic anomalies. In order to estimate the heading for an unconstrained smartphone, principal component analysis (PCA) of acceleration is applied to determine the offset between the orientation of smartphone and the actual heading of a pedestrian. Moreover, a particle filter with vector graph assisted particle weighting is introduced to correct the deviation in step length and heading estimation. Extensive field tests, including four contexts of carrying a phone, have been conducted in an office building to verify the performance of the proposed algorithm. Test results show that the proposed algorithm can achieve sub-meter mean error in all contexts.

## 1. Introduction

As one of the most challenging technologies in location-based services (LBS), indoor localization has generated great concern over the last decade. There are diverse indoor localization applications that widely exist in our daily life, such as context detection, health care, emergency events and so on [1]. However, compared to outdoors where global navigation satellite systems (GNSS) are essential and even dominating technologies, indoor localization encounters a series of challenges due to complex indoor environments, e.g., severe multipath effect, Non-Line-of-Sight (NLOS) conditions, high signal attenuations, and noise interferences. Therefore, a reliable indoor localization solution with high accuracy is still a gap of seamless navigation.

According to various basic measuring principles, localization method can be divided into four main categories: triangulation, direct sensing, pattern recognition, and dead reckoning [2]. Among these methods, triangulation-based and direct-sensing-based localization approaches need infrastructure assistance and depend on the deployment of beacons at known positions, e.g., Wi-Fi [3,4,5] and RFID [6,7]. Besides, some of these approaches are susceptible to being interfered in the indoor environment, such as ultrasound [8,9], infrared [10] and Bluetooth beacons [11,12]. Furthermore, the limitation of pattern recognition-based localization methods [13,14] requires tedious work load of model training, high storage capacity, and significant computing power. Pedestrian dead reckoning (PDR) localization technique [15,16,17], which utilizes the inertial sensors to estimate a pedestrian’s location with lower deployment cost and computation over other localization methods. Meanwhile, with the built-in sensors, smartphones have become really ubiquitous portable devices that provide not only communication services for people in their daily life but also personal localization functionalities [18,19,20,21,22].

PDR localization techniques, however, have a drawback in that they are only able to provide the required accuracy in a limited duration due to the sensor errors arising from random zero bias and oscillation noise. For low-cost inertial sensors especially, the accumulating errors grow rapidly with the travel distance of pedestrians. In order to mitigate this disadvantage, assisted localization solutions or additional information are demanded to correct the accumulating errors and achieve consistently high-precision indoor localization in PDR systems. For instance, GNSS is usually used for calibrating the PDR drifts outdoors in integrated systems [23,24,25]. Except GNSS, existing infrastructures, such as WLAN, ZigBee, RFID, camera and ultrasound, are utilized to provide indoor calibrations of PDR systems [26,27,28,29,30]. Taking account of the practical factors, such as high accuracy, low cost, and easy deployment, the above systems have various limitations. Alternatively, indoor maps can be utilized to constrain the pedestrian’s trajectory and improve the performance of PDR systems. Without the requirement of infrastructures, the indoor maps make self-contained navigation on a smartphone become feasible.

## 2. Motivation and Paper Outline

Recently, some researchers [31,32,33,34] investigate the pedestrian indoor localization methods which integrate inertial sensors with map information. Most researches with high performance rely on dedicated inertial measurement units (IMUs) which require fixed placement or a specific carrying context. Additionally, most of these methods only construct the trajectory of the user by detecting of turning events or path matching, which are prone to fail when the walking routes are intricate such as multi-turn paths or fail due to the symmetry of environment. In addition, the feedback mechanism of these methods used to improve the localization accuracy makes real-time implementation difficult.

In this paper, we present a reliable path-independent and real-time indoor localization method that relies on smartphone sensors and indoor vector graph. To tackle the drift problem of low-cost inertial sensors, we propose a robust step detection algorithm. Applying the local gravity value crossings detection and autocorrelation operation of measured acceleration signals, we detect steps with a low false rate. Then the step length is estimated based on the relationship between step length and step interval (reciprocal of step frequency), and the recognition of pedestrian motion patterns using smartphone sensors. Heading determination is accompanied through applying the principal component analysis (PCA) to infer the heading offset between the pedestrian and her smartphone, and gained from the yaw angle of the smartphone sensors. Moreover, magnetic anomalies, which influence heading determination while the attitudes of smartphones are acquired, are identified and avoided effectively by proposed two-phase filter.

Furthermore, a multi-dimensional particle filter algorithm, which fused information from PDR and an indoor vector graph, is designed to correct the sensor drifts and improve the localization accuracy by revising the errors introduced in step length estimation and heading determination. Therefore, each particle in our algorithm contains not only position information, but also step length and heading information of the pedestrian. In addition, to satisfy the demand of actual usability, vector graph is automatically transformed into grid constraint information and KNN (k-Nearest Neighbor) algorithm is adopted to accelerate the establishment of the lookup table. As a result, these procedures make the proposed system be able to operate in real-time on a smartphone. Reference [35,36] has briefly presented the preliminary results of our previous research on this topic.

In the rest of this paper, we introduce first the overall architecture of our indoor localization system in Section 3. After that, Section 4 presents the PDR algorithm employed in our work and it is followed by a detailed description of the algorithms for step detection, step length estimation and heading determination. As one main module, particle filter algorithm then is presented in Section 5. Afterward, the performance of the proposed algorithm is evaluated through an indoor field test conducted in our laboratory building in Section 6. Finally, the conclusion and future works are drawn in the last section.

## 3. Indoor Localization System Architecture

**Figure 1:**
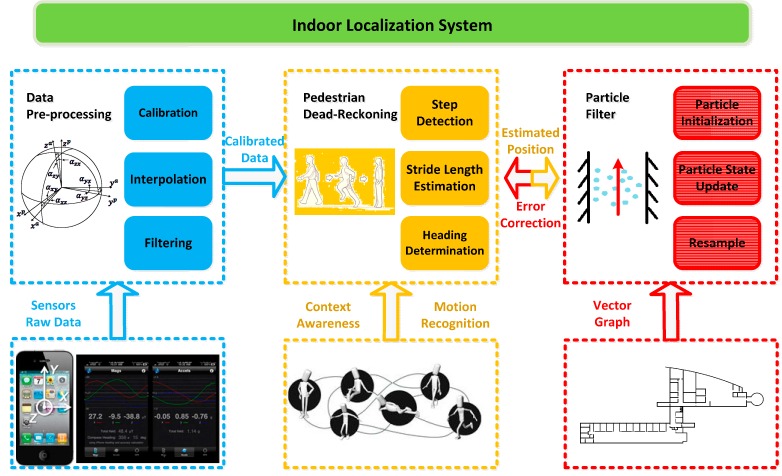
Overall system architecture.

As shown in Figure 1, the architecture of our indoor localization approach consists of three main modules: data preprocessing, PDR, and particle filter.

The data preprocessing module achieves the functionalities of calibrating, interpolating, and filtering the data from built-in sensors including accelerometer, gyroscope and magnetometer measurements. The processed data will be further utilized in the PDR module to calculate the location of a pedestrian. The calibration procedure is used to eliminate the zero offset and scale errors arise from low cost sensors. In practice, the sensor data are calibrated based on the fact that the magnitudes of sensors measurements are constant while the smartphone is kept still or rotated with an even angular rate [37]. To cope with low sampling frequency and unstable output of the smartphone sensors, raw sensor data are interpolated using cubic spline interpolation method [38]. At last, the sensor data are low-pass filtered using finite impulse response (FIR) filter to remove the high frequency noise.

After receiving the preprocessed data, the PDR module starts to estimate and update the position of a pedestrian step by step given an initial position obtained through GNSS, RFID and so on. For the sake of unconstrained smartphone applications, context-awareness for phone carrying and motion recognition is adopted in the PDR module to improve the accuracy of step length estimation.

In addition, the particle filter module is responsible for fusing the final localization results for external applications by iterative calculation and providing feedback information to correct the step length and heading error from PDR with the constraint condition of an indoor vector graph. With regard to the details of sub-modules of PDR module and the specific algorithms of particle filter module, they will be elaborated in the following sections.

## 4. Pedestrian Dead Reckoning

Pedestrian dead reckoning (PDR) is a relative navigation technique, which determines the relative location of a pedestrian by using step detection, step length estimation, and heading determination. Typically, the accelerometer measurements are utilized to implement step detection and step length estimation, and heading determination is simultaneously completed by fusing the information from gyroscopes, accelerometers, and magnetometers.

In the PDR system, the position of a pedestrian can be propagated as the following equations:

(1) $$\left\{ \begin{array}{l} x_{k+1}=x_{k}+SL_{k}\cdot \sin(\theta_{k^{*}})\\ y_{k+1}=y_{k}+SL_{k}\cdot \cos(\theta_{k^{*}}) \end{array}\right.$$

where *x* and *y* are the coordinates in north and east directions, *SL* is the step length and θ is the heading at moment k* when smartphone heading is close to pedestrian heading during a step. k* is not exactly same as subscript *k* that denotes the index of steps and details of k* definition are explained in Subsection 4.3. From Equation (1), it is shown that we can estimate the position of the pedestrian at any moment given an initial position and the step length and the heading of the pedestrian derived from sensors.

**Figure 2:**
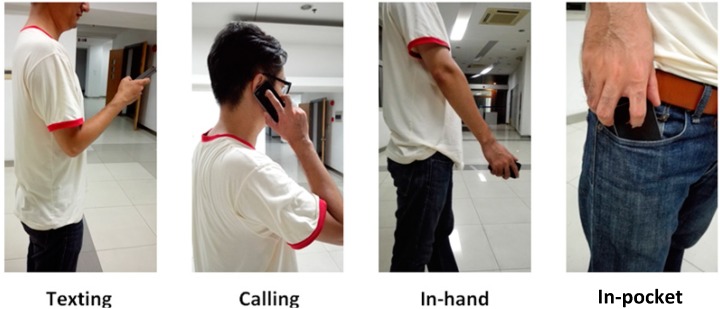
Different contexts of carrying a smartphone in daily use.

Most researches on PDR require fixing sensors on the body or holding the sensors in a certain gesture. However, a pedestrian carries a phone in diverse ways and uses a phone for different purposes. For instance, a pedestrian uses smartphone for texting, surfing the Internet, navigating, or making a call. The phone may be in hands or be placed in pocket. In this paper, four contexts of carrying a smartphone, as shown in Figure 2, are considered including Texting *C*_1_, Calling *C*_2_, In-hand *C*_3_ and In-pocket *C*_4_. It can be found through experiments that the algorithms and performances of a PDR system are closely linked to different contexts.

### 4.1. Step Detection

**Figure 3:**
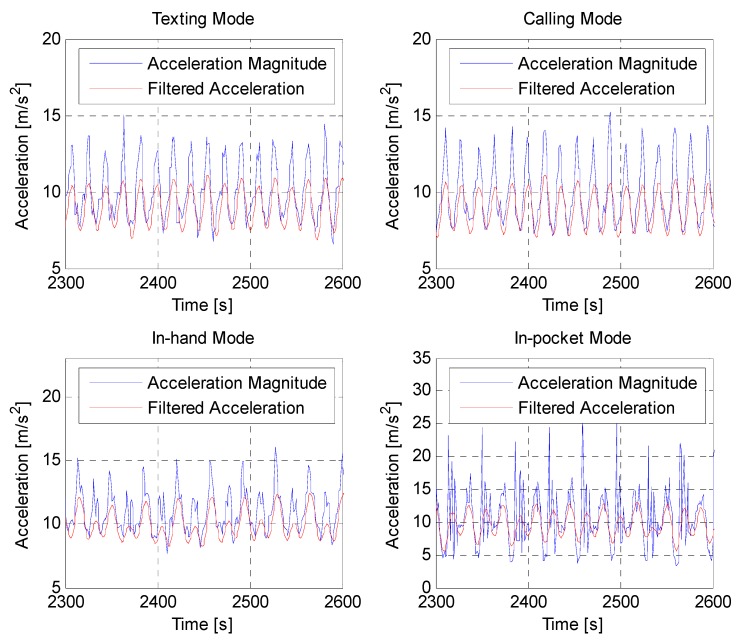
Acceleration magnitudes during normal walking in different carrying contexts and their corresponding filtered signals.

Step detection algorithm, as a fundamental of a PDR system, is crucial to influence the performance of the pedestrian navigation system. As mentioned previously, the accelerometer signal is usually employed to detect the steps of the pedestrian. In general, the output of accelerometer presents harmonic oscillation waveforms that result from walking behaviors [19]. Figure 3 shows the magnitude of total accelerations collected from a smartphone during normal walking in different carrying contexts.

As shown in Figure 3, the total acceleration magnitudes (blue lines) have approximately bimodal oscillation mode with interferences that arise from perturbations of the hand or shakes of the smartphone in the pocket. The high-frequency noises are filtered using a low-pass filter with a 3 Hz cut-off frequency and then the periodicity of the filtered accelerations (red lines) becomes more obvious. The magnitude *a_mag_* can be expressed as:

(2) $$a_{mag,k}=\sqrt{a_{x,k}^{2}+a_{y,k}^{2}+a_{z,k}^{2}}$$

where *a_x_*_,*k*_, *a_y_*_,*k*_ and *a_z_*_,*k*_ are the measurements from the triaxial accelerometer, whose respective directions as indicated in Figure 1. Unlike other approaches using vertical direction of the acceleration to detect steps [39,40,41], the total acceleration magnitude is used in our algorithm, which is insensitive to the orientation of a smartphone. Therefore, this variable can be adapted to different walking patterns.

**Figure 4:**
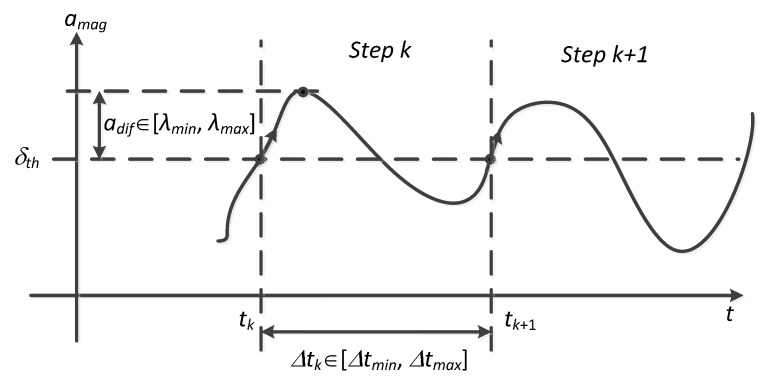
Identification of candidate steps.

According to the value of *a_mag_*, a candidate step at time *t_k_*, where *k* denotes the index of steps, is identified by following criteria (as shown in Figure 4):

C1. The total acceleration magnitude *a_mag_* has to cross the threshold δ*_th_* from negative to positive.
C2. The time interval ∆*t* between two consecutive steps defined by C1 must be within the interval threshold from ∆*t*_min_ to ∆*t*_max_.
C3. The difference *a_dif_* between extreme values of *a_mag_* during a step phase and the threshold δ*_th_* has to be among λ_min_ to λ_max_, otherwise a perturbation point is recorded.

In view of the irregular fluctuations of *a_mag_* due to various individual ways of walking, the threshold δ*_th_* in C1 is updated dynamically according to the mean value of *a_mag_* over a step period, *i.e.*,

(3) $$\delta_{th}=\frac{1}{\Delta t_{k}}\int_{t_{k}}^{t_{k+1}}a_{mag}(t)dt$$

**Figure 5:**
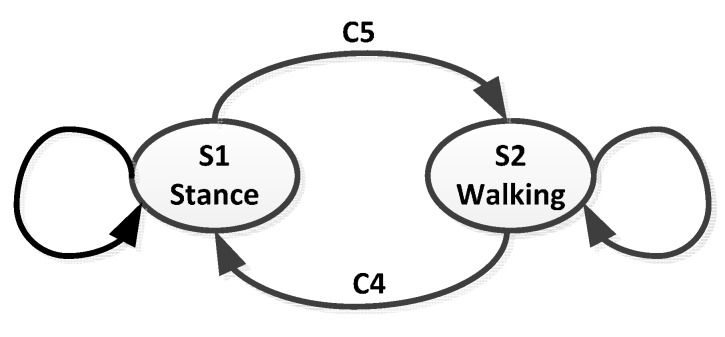
Finite state machine (FSM) for coping with carrying modes transformation during actual walking.

In order to distinguish walking motions from other pedestrian activities, a finite state machine (FSM) with two states, S1 *Stance* and S2 *Walking*, is defined. The state transitions of the FSM are determined by the autocorrelation of *a_mag_* and candidate step counts within a sliding window of the length *T_s_*. The criteria of state transitions are given as follows (see as Figure 5):

C4. Transition from S2 to S1: there is no candidate step or there exist perturbation points in the sliding window.
C5. Transition from S1 to S2: the candidate step number is more than one and there is not any perturbation point within the sliding window, meanwhile the autocorrelation value *r_mag_* is larger than threshold *r_th_*.

Note that the autocorrelation mentioned in C5 does not need to be computed if the state maintains S2 *Walking* when the candidate steps become valid steps. The purpose of this scheme is to effectively reduce unnecessary computations and thus save power consumption.

### 4.2. Step Length Estimation

**Figure 6:**
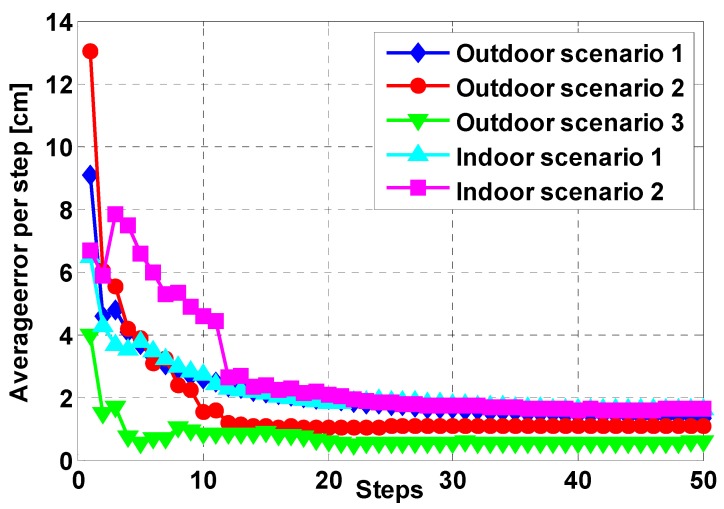
Step length estimation results of proposed training method in different scenarios.

In the PDR system, step length estimation, combined with heading determination, is utilized to compute the traveled distance and update the position of a pedestrian on condition that the previous position is known. As described in IMU based PDR systems, the step length of a pedestrian is not constant and varies with walking speed, step frequency, acceleration variance and so on [42,43,44]. In order to estimate the travel distance of a pedestrian accurately, adaptive step length estimation must be adopted according to these variations. Generally, the step length is estimated using a linear combination of step frequency and acceleration variance through following equations:

(4) StrideLength L=α⋅fre+β⋅var+γ

where *fre* is step frequency, *var* is acceleration variance during one step; α and β are weighting factors of step frequency and acceleration variance; γ is a constant. For different pedestrians, the model parameters α, β, γ are different and usually required by offline training. However, to accelerate training process and realize near-real-time training, an optical flow based step length model training method is proposed in our work [45]. By establishing the relationship between optical flow and velocities of a pedestrian as Equation (5), the step length model parameters can be trained every step and thus the training process can be completed within twenty steps as shown in Figure 6. Therefore, the efficiency of our proposed method is obviously higher than conventional methods as [46], where hundreds of meters distance is needed to train model parameters.

(5) $$\left\{ \begin{array}{l} x=\frac{f_{y}(u-c_{x})}{f_{x}(v-c_{y})\sin\phi +f_{x}f_{y}\cos\phi }\cdot h\\ y=\frac{(v-c_{y})\cos\phi -f_{y}\sin\phi }{(v-c_{y})\sin\phi +f_{y}\cos\phi }\cdot h \end{array}\right.$$

where *f_x_*, *f_y_*, *c_x_* and *c_y_* are intrinsic parameters of the smartphone camera, and they denote focal length and principal point respectively. *h* and ϕ are the distance and angle between the smartphone and ground.

The step frequency and acceleration variance in Equation (4) are obtained as:

(6) $$\left\{ \begin{array}{l} fre_{k}=1/\left(t_{k}-t_{k-1}\right)\\ var_{k}=\sum_{t=t_{k-1}}^{t_{k}}\frac{{\left(a_{k}-{\bar{a}}_{k}\right)}^{2}}{n} \end{array}\right.$$

where *frek* and *vark* are step frequency and acceleration variance at *tk* respectively; *tk* means timestamp of the step *k*; *ak* is acceleration signal and ${\bar{a}}_{k}$ is average acceleration during the step; *n* is the number of sensor samples.

**Figure 7:**
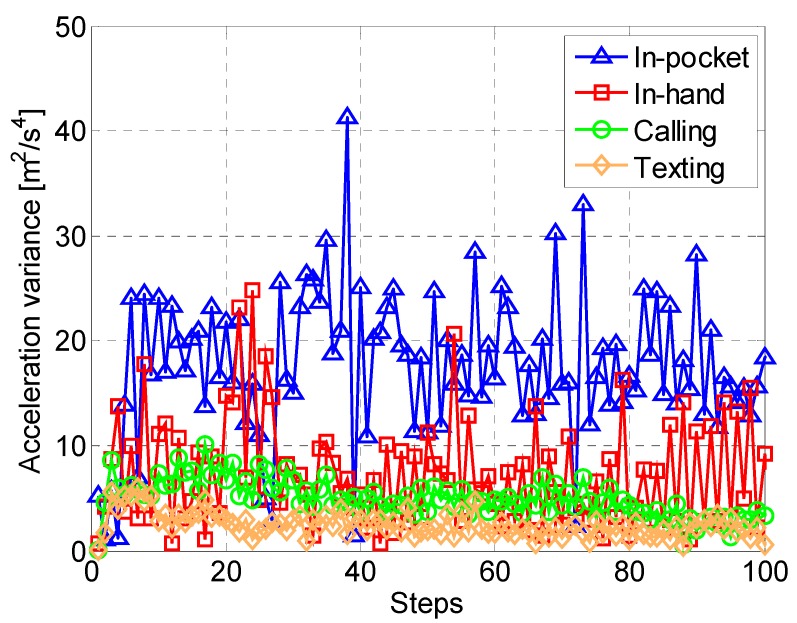
Acceleration variances in different contexts of carrying a smartphone.

The acceleration variance *var_k_*, however, varies widely depending on different smartphone carrying contexts as shown in Figure 7. In addition, the step length is also influenced by different motion states. Therefore, an adaptive step length estimation algorithm is needed with the consideration of various carrying contexts and motion states of a smartphone user.

According to kinetic characteristics, four motion states are considered in our approach: static state *S*1, walking state *S*2, turning state *S*3 and stairs state *S*4 [36]. In order to distinguish different contexts of carrying a smartphone, the mean value of gravity $\bar{G}=\{{\bar{g}}_{x},{\bar{g}}_{y},{\bar{g}}_{z}\}$ and its projections $\tilde{G}=\{{\tilde{g}}_{xy},{\tilde{g}}_{xz},{\tilde{g}}_{yz}\}$ on each axis and planes are chosen as the features as following equations:

(7) $$\left\{ \begin{array}{l} {\bar{g}}_{\eta }=\frac{1}{\tau }\int_{t_{k}-\tau }^{t_{k}+\tau }g_{\eta }(t)dt,\text{ (}\eta =x,y,z)\\ {\tilde{g}}_{\mu \nu }=\frac{1}{\tau }\int_{t_{k}-\tau }^{t_{k}+\tau }\sqrt{g_{\mu }^{2}(t)+g_{\nu }^{2}(t)}dt,\text{ (}\mu =x,y,z;\;\nu =x,y,z;\;\mu \neq \nu ) \end{array}\right.$$

where *t_k_* is the timestamp of steps as defined above and τ is the length of features sampling window.

Moreover, to classify motion states, the features of smartphone sensor signals are extracted in time and frequency domains respectively, including amplitudes, mean values, variances, *etc.* defined in [22], and then different motion states are recognized by means of Random Forests (RF) algorithm [47].

**Figure 8:**
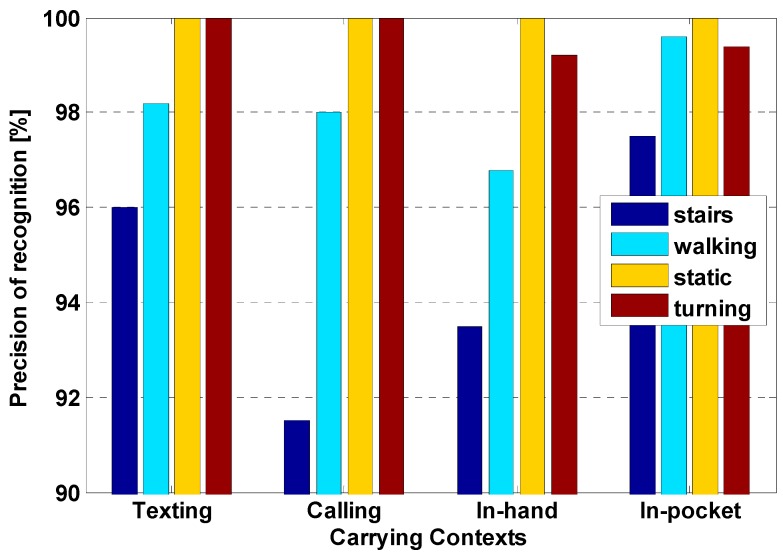
Precision of motions recognition in different contexts of carrying a smartphone.

The confusion matrix for the carrying contexts recognition from proposed gravity-based classifier is listed in Table 1 and the precision of motions recognition in different contexts of carrying a smartphone is shown in Figure 8. It is noted obviously that different motion states can be classified with high accuracy, which enables our PDR approach adaptively to estimate the step length with different model parameters as Equation (8).

(8) $$L_{S}=\alpha_{S_{i},C_{j}}\cdot fre+\beta_{S_{i},C_{j}}\cdot var+\gamma_{S_{i},C_{j}},\;(i,j=1,2,3,4)$$

where α*_Si_*_,*Cj*_, β*_Si_*_,*Cj*_, γ*_Si_*_,*Cj*_ are adaptive model parameters depending on different motion states *S_i_* and carrying contexts *C_j_*. The parameters satisfy following conditions:

• ${\text{α}}_{S_{1},C_{j}}={\text{β}}_{S_{1},C_{j}}={\text{γ}}_{S_{1},C_{j}}=0,\;\forall j$, *i.e.*, step length is zero when a pedestrian is static;
• ${\text{α}}_{S_{2},C_{j}}\geq {\text{α}}_{S_{3},C_{j}},{\text{β}}_{S_{2},C_{j}}\geq {\text{β}}_{S_{3},C_{j}},\;\forall j$, *i.e.*, step length at turning points is usually smaller than a pedestrian is walking straightly;
• ${\text{α}}_{S_{4},C_{j}}={\text{β}}_{S_{4},C_{j}}=0,\;{\text{γ}}_{S_{4},C_{j}}=h_{sidestep},\;\forall j$, *i.e.*, step length is a constant equal to the height of sidesteps when a pedestrian is climbing stairs;
• ${\text{β}}_{S_{i},C_{1}}\geq {\text{β}}_{S_{i},C_{2}}\geq {\text{β}}_{S_{i},C_{3}}\geq {\text{β}}_{S_{i},C_{4}},\;\forall i$, *i.e.*, the model parameters β*_Si_*_,*Cj*_ are inversely proportional to the acceleration variances in different carrying contexts. For example, the acceleration variance is the largest in In-pocket context *C*_4_ usually, thus the model parameter β*_Si,C_*_4_ is smaller than the parameters in other contexts.

Therefore, for each pedestrian, the model parameters are all needed to be trained to cope with different motion states and carrying contexts. After the training stage, we can adaptively select appropriate model parameters to estimate the pedestrian’s step length according to the corresponding motion state and carrying context. Additionally, the model parameters are further adjusted online using constraint information derived from a vector graph, which will be investigated in Section 5.

**Table 1:** Confusion matrix for the carrying contexts recognition (Unit: %).

Carrying Contexts	Recognized Carrying Contexts			
	Texting	Calling	In-Hand	In-Pocket
**Texting**	100	0	0	0
**Calling**	0	98.99	0	1.01
**In-hand**	0	0	100	0
**In-pocket**	0	0	0	100

**Figure 9:**
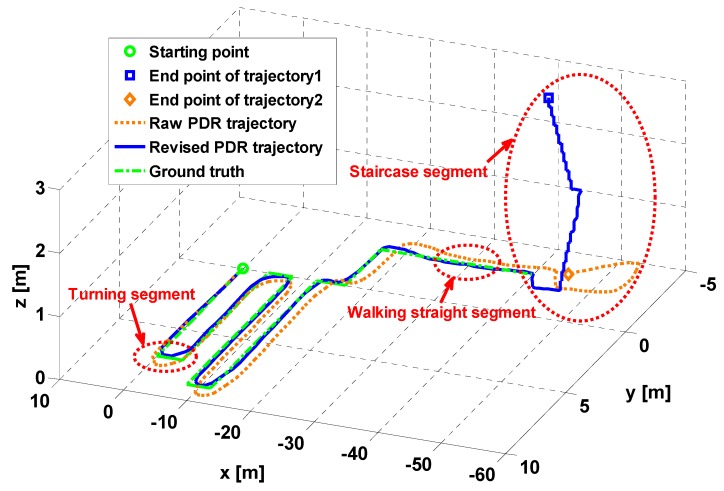
Illustration of proposed adaptive step length estimation for revising a pedestrian’s trajectory in PDR algorithm.

In general, it is a challenge for PDR algorithms in the complicated routes with multiple turnings and staircases. In view of this situation, an experiment containing different motion states was conducted to test our proposed method of adaptive step length estimation described above. Figure 9 demonstrates two trajectories of the test: yellow dotted line represents raw trajectory using conventional PDR and blue solid line represents revised trajectory using proposed method. Ground truth is denoted by green dashed line. Three segments, marked by red ellipses, indicate three typical scenarios with different motion states including turning, walking and using stairs. The turning segment shows that the positioning accuracy is improved with revisions of step length by applying the proposed algorithm. Moreover, in the staircase segment with proposed solution, 3D positioning of a pedestrian can be realized with the recognized motion state of using stairs. However, the raw PDR trajectory using conventional PDR can only provide 2D locations.

### 4.3. Heading Determination

Heading determination is one of the most challenging parts of a PDR system, because the error of heading leads to a fast growth of localization error. As the main reason that degrades localization accuracy, several researchers have pointed out that unbounded heading error could be more than 40° [48]. Usually, attitude and heading reference system (AHRS) algorithm fuses data from magnetic angular rate and gravity (MARG) sensor arrays (including accelerometers, gyroscopes and magnetometers) to acquire the smartphone sensor’s attitude represented in Euler angle or quaternion. Gyroscope, among MARG, is the main error source and its random error needs to be corrected periodically with magnetic measurements to update the yaw angle. In an indoor environment, however, the magnetometer may be severely disturbed by the soft iron from modern buildings. Thus, reliable heading determination is difficult to achieve. Moreover, for indoor localization on a smartphone, there is another problem that the yaw angle of the smartphone may be different from the pedestrian’s heading regarding various carrying modes. Therefore, two tasks need to be done to solve above problems before we implement heading determination. One is to mitigate the magnetic influence. The other one is to infer the offset between the calculated heading from smartphone sensors and the actual heading of a pedestrian. In common with step length model parameters, heading offsets are also corrected constantly in particle filter algorithm which will be detailed in Section 5.

**Figure 10:**
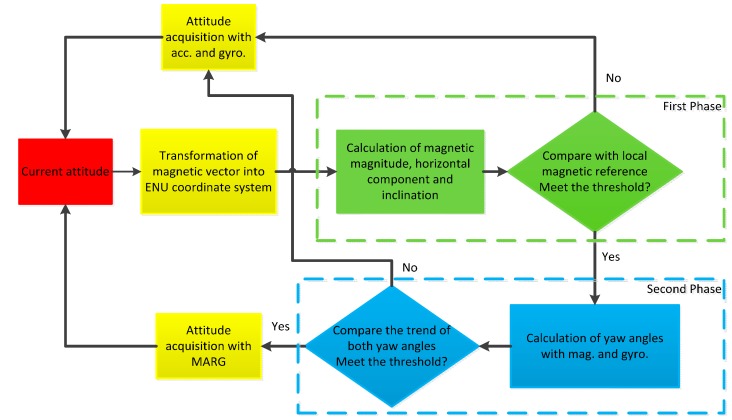
Flowchart of proposed two-phase filter scheme for avoiding magnetic anomalies.

To acquire the reliable attitude of the smartphone free from the distortion of magnetic anomalies, we have to distinguish the trustable magnetic readings from the abnormal ones. A two-phase filter scheme is implemented in our work and Figure 10 shows the flowchart of the attitude acquisition algorithm. As shown in Figure 10, in the first phase, features of magnetic field including magnitude, horizontal component and inclination are compared with international geomagnetic reference field (IGRF) [49]. If the differences between the features and their reference values are within the thresholds, the algorithm enters the second phase. Afterwards, the similarities in trend between yaw angles from 9DOF (degree of freedom) and 6DOF (without magnetometer) fused AHRS methods are compared using Euclidean distances defined as:

(9) $$ED_{yaw}=\sqrt{\sum_{k=n}^{n+N-1}{\left[(\psi_{k}^{9DOF}-{\bar{\psi }}^{9DOF})-(\psi_{k}^{6DOF}-{\bar{\psi }}^{6DOF})\right]}^{2}}$$

with

(10) $${\bar{\psi }}^{9DOF}=\frac{1}{N}\sum_{k=n}^{n+N-1}\psi_{k}^{9DOF}\text{ and }{\bar{\psi }}^{6DOF}=\frac{1}{N}\sum_{k=n}^{n+N-1}\psi_{k}^{6DOF}$$

where *N* is the size of sliding window; ${\bar{\psi }}^{9DOF}$ and ${\bar{\psi }}^{6DOF}$ are the mean values of yaw angles from different fused AHRS methods in the sliding window. If the distance *ED_yaw_* meets the threshold condition, 9DOF fused AHRS method is adopted, otherwise 6DOF method is adopted.

**Figure 11:**
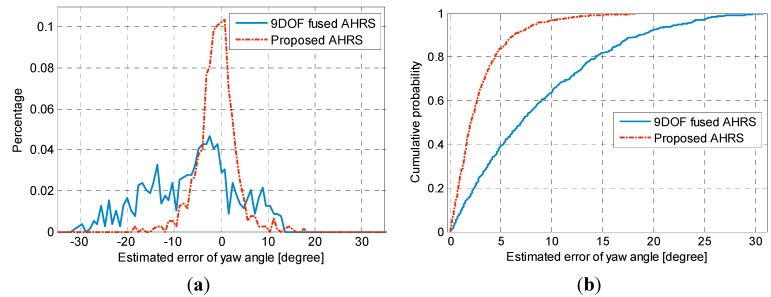
(**a**) Probability density functions of estimated errors in yaw angles; (**b**) Cumulative probability functions of estimated errors in yaw angles.

Figure 11 shows the probability function and cumulative probability function of errors between the estimated yaw angle using the proposed algorithm and the true yaw angle when the pedestrian walked along the appointed trajectory with a closed loop. It can be found that the error distribution of 9DOF fused AHRS method is between −30° and 25° and its mean value is negative, which brings large errors in positioning. By efficiently filtering magnetic anomalies, the errors of estimated yaw angles are limited within −10° to 10° using the proposed AHRS method and the range over which 90% of the yaw angle errors occur is reduced from 18.6° to 6.2°.

In consideration of smartphone carrying contexts of a pedestrian, four smartphone carrying modes, as shown in Figure 2, are defined in our research. For Texting and Calling modes, the yaw angles of the smartphone sensors are relatively stable, thus the heading offset almost remains constant and the heading can be determined at any time once the offset is known. Nevertheless, for In-hand and In-pocket modes, the yaw angles change dynamically even the pedestrian is walking straight. In order to determine the heading in dynamic states, we take advantage of the characteristics of walking behaviors.

**Figure 12:**
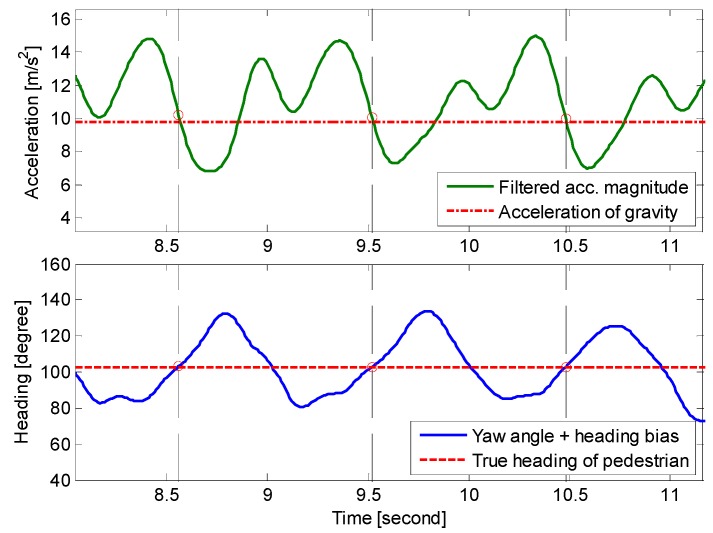
True heading of pedestrian acquired based on acceleration magnitude characteristics during walking.

As shown in Figure 3, the smartphone inertial sensors attached to a pedestrian will produce periodic acceleration magnitude signals during walking. The magnitude of acceleration is not only useful for detecting steps but also effective to determine a pedestrian’s heading, especially when the smartphone is in the pocket or swinging with the hand. During each signal period, the moment denoted as k* in Equation (1), can be observed when the acceleration magnitude crosses the gravity. At that time, the heading offset between the smartphone and a pedestrian is approximately the same as the moment when the pedestrian is at standstill. In general, the offset can be known by detecting the duration when the smartphone screen is facing the pedestrian. This duration is almost available because the pedestrian needs to start the application and select personal positioning parameters such as initial position and vector graph database. As shown in Figure 12, despite of the dynamic changing of smartphone sensors’ yaw angles, the moment to access the true heading can be determined by the time when the magnitude of acceleration crosses the threshold of gravity.

**Figure 13:**
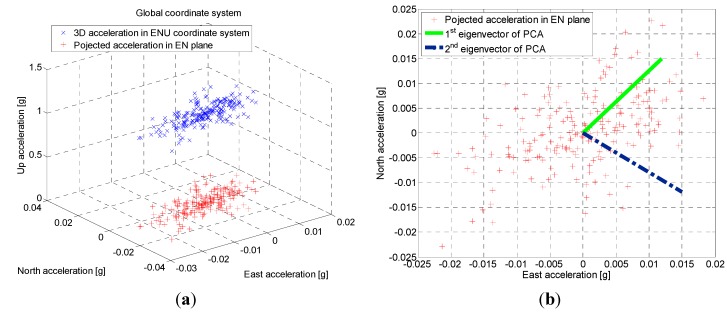
Illustration of heading determination by employing principal component analysis (PCA): (**a**) Description that three-dimensional acceleration is projected to global horizontal plane; (**b**) Eigenvectors extraction from the acceleration of horizontal plane to determine the heading of a pedestrian.

Compared to other modes, it is hard to obtain the offset using above method in the In-pocket mode due to a higher degree of freedom. It has been observed that the largest variations in the horizontal plane of the acceleration signal are parallel to the direction of motion while walking. Thus, the heading of a pedestrian can be determined by principal component analysis (PCA) of acceleration components. To apply PCA to acquire the heading of a pedestrian, the acceleration in sensor frame is first rotated to global East-North-Up (ENU) frame using the present attitudes of the smartphone sensor. A low-pass filter then is utilized to eliminate jitters in the acceleration. Finally, as shown in Figure 13, PCA is employed to the horizontal acceleration components to determine the heading as the direction of the first eigenvector.

**Figure 14:**
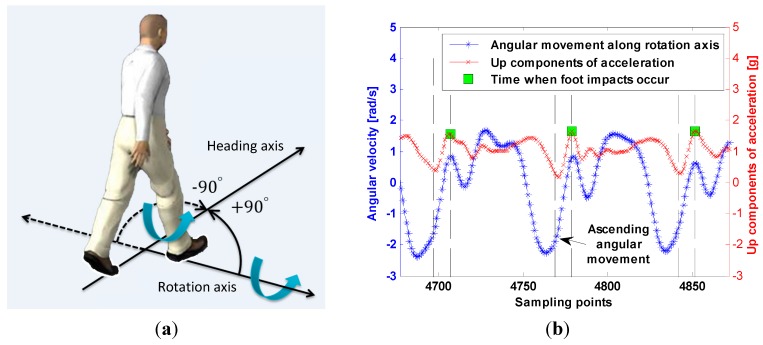
Illustration of 180° ambiguity elimination: (**a**) Description of rotation before a foot impacts with the ground; (**b**) Motion features mined from up acceleration components and angular movements are utilized to eliminate ambiguity.

However, the heading acquired from PCA has the problem of 180° ambiguity [50]. Motion features mined from accelerometer and gyroscope measurements can help solve the problem of ambiguity as illustrated in Figure 14. When the smartphone is placed in the pocket, each short period before foot impacts with the ground, the leg keep swinging forward and the smartphone is rotating. Based on the above fact, there should be a positive rotation along solid line direction (or a negative rotation along dotted line direction) of rotation axis in a right-handed coordinate system as shown in Figure 14a. Therefore, we can determine the pedestrian heading by examining the trend of angular movement along rotation axis before the foot impacts as shown in Figure 14b. If the angular movement is ascending, pedestrian heading is gained by adding 90° to the acquired heading from PCA. Conversely, if the angular movement is descending, pedestrian heading is gained by subtracting 90° from the acquired heading.

**Figure 15:**
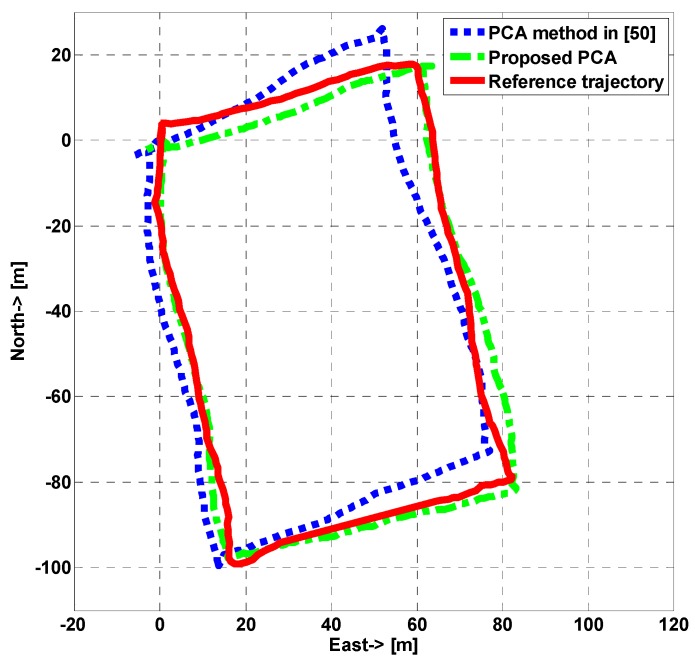
Estimated trajectories using different PCA methods.

Different from the method in [50], a variable-size window is adopted in our PCA approach. The window size depends on the time interval between two consecutive foot impacts points. Since the smartphone is put in one side pocket, the foot on the side with smartphone impacts on the ground once every two steps. Given variable step frequencies in normal walking, it is better to apply a variable-size window in PCA instead of a fix-size window which is hard to cover two steps exactly. For comparison, an experiment situated at an outdoor football stadium was performed. Figure 15 shows the estimated trajectories using two different PCA methods and the reference trajectory was gained from GPS. The experimental result indicates that our proposed PCA method can improve the positioning accuracy due to better heading determination.

## 5. Particle Filter Algorithm

Particle filter is a sophisticated model estimation algorithm based on simulation to deal with system with non-Gaussian noise. The proposed algorithm in this paper fuses status updates from PDR and constraints from an indoor vector graph to acquire the posterior distribution of a pedestrian’s location. Multidimensional particles are used that include position coordinates, estimated parameters of step length and heading. The PDR propagation model can be learned and corrected during the operation of the proposed particle filter algorithm. To save the computational cost in intersection detection, map construction and optimization of the vector graph are implemented.

### 5.1. Particle Filter Implementation

In our proposed particle filter algorithm, objects in vector graph including walls and furniture are chosen as constraints to limit a pedestrian’s trajectory. Consequently, binary particle weight is implemented and defined as: one indicates the particles are alive *i.e.*, their trajectories intersect with nothing; whereas zero indicates the particles are dead *i.e.*, their trajectories intersect with any object in the vector graph. Since normalization of the weights is unnecessary and resampling also becomes more efficient, the heavy computation of particle filter algorithm can be simplified by adopting binary particle weight.

**Figure 16:**
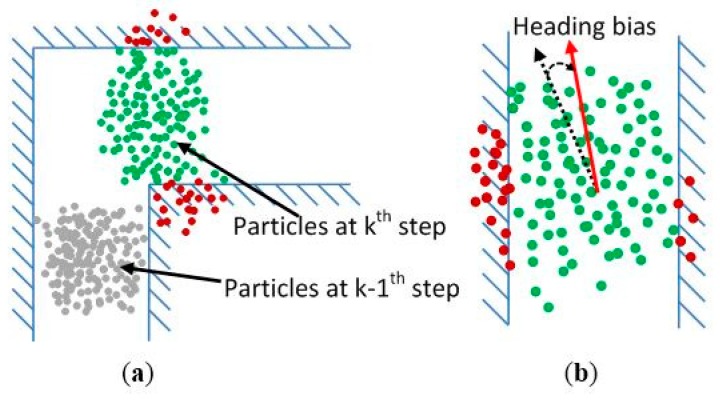
Illustrations of particles status in (**a**) turning situation and (**b**) non-turning situation. Red points indicate dead particles and green points indicate surviving particles.

As shown in Figure 16, it can be found that errors of heading determination usually make the particles hit obstacles in the vector graph when a pedestrian walks straight while errors of step length estimation mostly cause the particles to die when the pedestrian turns. Compared to particles with only position information, each particle in our algorithm also contains step length model parameters and heading bias information. The state of a particle is defined as:

(11) $$S_{k}^{i}=\left\{x_{k}^{i},y_{k}^{i};x_{k-1}^{i},y_{k-1}^{i};\alpha_{k}^{i},\beta_{k}^{i},\gamma_{k}^{i};\varepsilon \theta_{k}^{i}\right\},\;i=1,2,\ldots ,N.$$

where *N* is the number of particles; $x_{k}^{i}$ and $y_{k}^{i}$ are the position information of the *i^th^* particle at the *k^th^* step; $x_{k-1}^{i}$ and $y_{k-1}^{i}$ are the position information at the former step; $\alpha_{k}^{i}$, $\beta_{k}^{i}$, and $\gamma_{k}^{i}$ are the step length model parameters as defined in Equation (4); ε$\theta_{k}^{i}$ is the noise of heading. The step length model parameters are updated and adaptive learned in the resampling phase according to the following rules:

• If the particles hit an obstacle in non-turning situation (heading difference is less than 25°), the step length model parameters of them are retained and can be inherited by the next generation particles;
• If the particles die in turning situation (heading difference is more than 25°), their model parameters are abandoned and only the model parameters of surviving particles are retained for generating new particles.

Although consideration has been taken to eliminate the effect of magnetic anomalies as described in Subsection 4.3, severe magnetic distortion in the indoor environment may still results in deviation in heading estimations. Extra heading correction can be achieved by investigating the heading of surviving particles as following:

(12) $$\theta_{k}^{bias}=\kappa \cdot \frac{1}{N_{s}}\sum_{i=1}^{N_{s}}\varepsilon \theta_{k}^{i}$$

where $\theta_{k}^{bias}$ is the heading bias at *k^th^* step; *N_s_* is the number of surviving particles; κ is the gain factor, which is used for controlling the degree of heading correction. Normally, $\theta_{k}^{bias}$ is close to zero since the noise ε$\theta_{k}^{i}$ is drawn from zero-mean Gaussian distribution. When the estimated heading is deviated from the true heading, the heading bias of surviving particles $\theta_{k}^{bias}$ is not equal to zero and it is used to correct the heading of next generation particles in the propagation phase.

**Figure 17:**
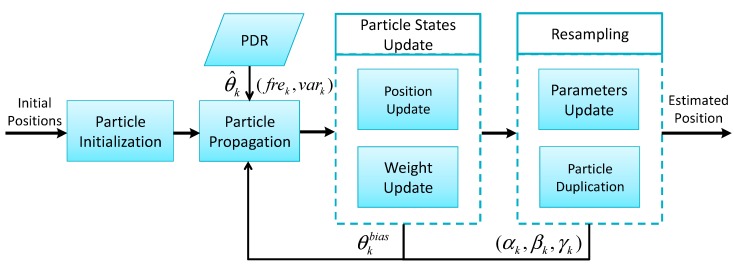
Flowchart of the proposed particle filter algorithm and its correction process.

The procedure of our proposed particle filter that fuses the position information from PDR and vector graph information is shown in Figure 17. In the phase of particle initialization, the positions of particles are initialized by pedestrian with additional zero mean Gaussian noise. The attributes of particles are set including particle number, particle weights, initial positions, standard deviation of step length and heading calibration, initial step length model parameters and thresholds. The positions of particles propagate as Equation (1). The estimated heading ${\hat{\theta }}_{k}$ and calculated step frequency *fre_k_* and acceleration variance *var_k_* are obtained from PDR to update particles’ positions. During particles propagation, the step length and heading of the *i^th^* particle are defined as following:

(13) $$\left\{ \begin{array}{l} SL_{k}^{i}={\text{α}}^{i}\cdot freq_{k}+{\text{β}}^{i}\cdot var_{k}+{\text{γ}}^{i}+\text{ε}SL_{k}^{i}\\ {\text{θ}}_{k}^{i}={\hat{\text{θ}}}_{k}+{\text{θ}}_{k}^{bias}+\text{ε}{\text{θ}}_{k}^{i} \end{array}\right.$$

where ε$SL_{k}^{i}$ and ε${\text{θ}}_{k}^{i}$ are artificially added white noise with a normal distribution. The weights of particles are then updated using the vector graph constraints. For any particle, whenever the connecting line from the former position to the current position intersects with objects in the vector graph, the trajectory is identified as invalid path and thus the particle’s weight is set to zero. In the resampling phase, the step length model parameters are retained or updated based on whether particles are turning. When the effective particle number is less than a preset threshold, surviving particles are randomly selected and duplicated to compensate the losing *N* − *N_s_* particles. Finally, the centroid position of particles is chosen as the estimated position at the present moment.

### 5.2. Map Construction and Optimization

**Figure 18:**
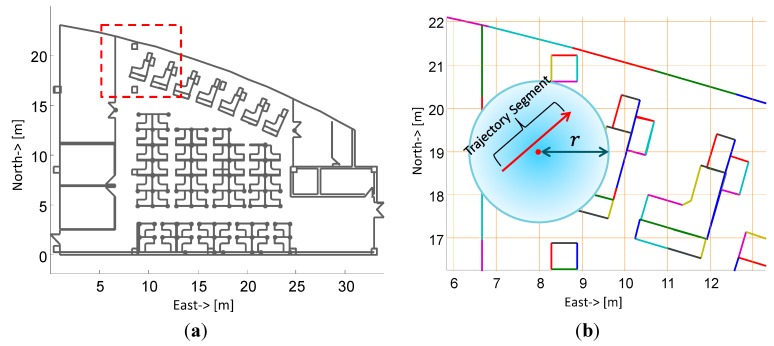
(**a**) The vector graph of an office building; (**b**) enlarged vector graph of marked box with red dotted line in (**a**) and the grids definition. Different segments of the vector graph are denoted by different colors.

The vector graph used in our algorithm, as shown in Figure 18a, is gained from the floor plan of an office building in the form of a DXF (Drawing Exchange Format) file, which contained various entities including segments, polylines and arcs. To simplify the computing, we transform polylines and arcs into segments artificially and establish a database to store all the entities. As defined above, the state $S_{k}^{i}$ of a particle contains last position ($x_{k-1}^{i}$, $y_{k-1}^{i}$) and current position ($x_{k}^{i}$, $y_{k}^{i}$), which means the trajectory from *k* − 1 step to *k* step is the segment connecting two positions. To accomplish the update of a particle’s weight, we have to determine whether its trajectory segment intersects with segments in the vector graph. In the worst cases, if the trajectory segment does not intersect with any segment (*i.e.*, the particle hits nothing), the time complexity of intersecting detection is *o*(*n_m_*) where variable *n_m_* is the number of segments in the vector graph. Therefore, it is computationally intensive to update all particles’ weights at each step.

**Figure 19:**
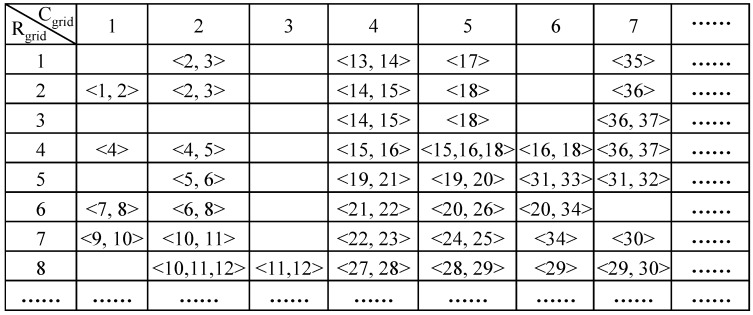
Established lookup table of grid points in the vector graph.

Since the step length of a pedestrian is limited, the trajectory segment is impossible to intersect with those segments that are far from the particles and thus it is unnecessary to detect all the segments of the vector graph for the weights update. Based on this fact, a spatial filter is designed. We divide the vector graph into uniform grids as shown in Figure 18b. The index numbers of segments that are within range *r* from a grid point are recorded and a lookup table is established as shown in Figure 19. In the table, each cell corresponds to a grid point and stores the index number of all potential segments which intersect with the trajectory segment at this grid point. As a result, it only needs to check several segments instead of all segments to complete intersecting detection. The computational cost is greatly reduced and it becomes independent of the size of vector graph.

Understandably, the shorter range *r* is, the fewer segments are involved. However, too small range *r* will lead to filter out the correct candidates of segments. Therefore, we define the minimum range *r* for searching candidates of segments in Equation (14). Assume that the midpoint of a trajectory segment is exactly at one of grid points, and then the search distance is equal to the half of maximum step length. Moreover, the largest range between the midpoint of a trajectory segment and the nearest grid point is equal to the diagonal of a grid, thus the minimum of *r* is described as:

(14) $$r_{\min}=\frac{SL_{\max}}{2}+\frac{L_{grid}}{\sqrt{2}}$$

where *SL*_max_ is the maximum step length of a pedestrian ; *L_grid_* is the side length of defined square grids. Consequently, we can infer the index number of a grid from the midpoint position as:

(15) $$\left\{ \begin{array}{l} R_{grid}=\left[\frac{x_{m}}{L_{grid}}+0.5\right]+1\\ C_{grid}=\left[\frac{y_{m}}{L_{grid}}+0.5\right]+1 \end{array}\right.$$

where *R_grid_* and *C_grid_* are row index number and column index number of the grid respectively; (*x_m_*, *y_m_*) is the position of the midpoint; [**·**] is rounding operation. KNN algorithm is adopted to accelerate the searching of the nearest segments from each grid point and the lookup table is established after de-duplication.

**Figure 20:**
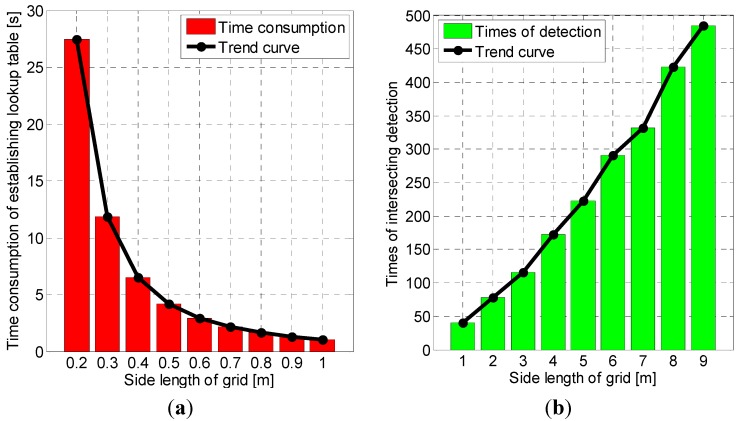
Illustrations of relationships between side length of grids and (**a**) time consumption of establishing the lookup table (**b**) times of intersecting detection for each particle. Black curves indicate the trend of variations.

For further illustration, the relationships between time consumption of establishing the lookup table, times of intersecting detection for each particle and side length of grids constructed in our algorithm are shown in Figure 20. With growth of side length of grids, time consumption of establishing the lookup table decreases from 28 s to 1 s as Figure 20a. However, average number of times in intersecting detection for each particle, meanwhile, rise from 40 to 485 as Figure 20b. Given the factor that the lookup table only needs to be established once and hundreds of particles need to update the weight, the computational cost can be further reduced by optimizing the grid size according to the size of experimental area and the number of particles.

## 6. Indoor Localization Field Test

In this section, to verify the performance of proposed indoor localization method in practical situations, a field test with four carrying modes of the smartphone was performed.

### 6.1. Field Test Setup

The test site was situated at an office building which is an 18 m × 12 m area as shown in Figure 18a and the total length of one lap is about 60 m. The obstacles in the map are consisted of walls, pillars, and computer desks. In the field test, an iPhone 4 was utilized and the output rate of the smartphone was set to 32 Hz. The output sensor data were calibrated and the output rate of the data was increased to 64 Hz by interpolation when proposed system was in operation. Three subjects, one female and two males, participated in the test. Participants walked ten rounds along the ground truth naturally with four smartphone carrying modes as shown in Figure 2.

### 6.2. Field Test Results

**Figure 21:**
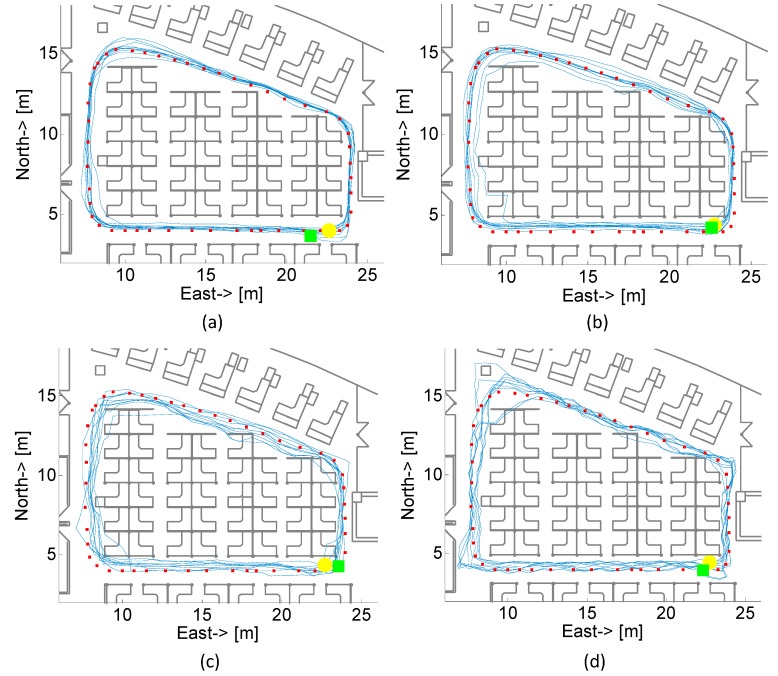
Estimated real-time trajectories of the pedestrian with different smartphone carrying modes: (**a**) Texting; (**b**) Calling; (**c**) In-hand; (**d**) In-pocket.

Figure 21 shows the estimated trajectory of the pedestrian with different smartphone carrying modes using proposed indoor localization method. The blue lines represent pedestrian’s trajectory and red squares represent ground truth that is demarcated by using a laser rangefinder. Yellow circle and green square denote starting point and end point, respectively. They are almost overlapping due to the high precision localization result. The result indicates that the positioning accuracy of our algorithm is reliable and accurate. It can be found from the figure that some trajectories walk through obstacles, which is due to part of deviate particles are still alive and the estimated position is derived from the centroid of all surviving particles at current moment.

Table 2 lists the detailed localization accuracy of the tests in four scenarios. We can find that the best result is Texting situation, the 50% percentile error is 0.51 m and 95% percentile error is 0.8 m; while the worst result is In-pocket situation, the 50% percentile error is 0.74 m and 95th percentile error is only 1.71 m. In comparison, the localization accuracy of the method without vector graph assistance is also listed in Table 2. It is obvious that the localization accuracy is improved with assistance of vector graph in our proposed method for all scenarios.

**Table 2:** Localization error from the proposed method and the method without vector graph assistance with four carrying modes (Unit: m).

Carrying Modes	Real-Time Localization Errors			
	50% Error		95% Error	
	Proposed	w/o Assistance	Proposed	w/o Assistance
**Texting**	0.51	0.93	0.80	2.24
**Calling**	0.45	1.14	0.88	4.54
**In-hand**	0.50	1.38	1.01	3.65
**In-pocket**	0.74	1.40	1.71	3.74

## 7. Conclusions

In this paper, we have presented an improved indoor localization method based on self-contained sensors of a commercial off-the-shelf smartphone. This method allows us to implement high-precision indoor positioning by integrating PDR algorithm and vector graph information without fixing a phone on the body. Field test results demonstrate that the proposed method, which is applicable to diverse carrying modes of smartphones, could be a promising scheme to be deployed into various mobile devices and IMUs based pedestrian localization systems. For future work, we plan to improve the algorithm in all aspects of PDR technologies especially heading determination, which is still an unsolved problem in complex indoor environment. In addition, other indoor localization technologies, such as Wi-Fi or RFID (Bluetooth), can be integrated into the current system to further improve the localization accuracy.
